# Connectedness among herds of beef cattle bred under natural service

**DOI:** 10.1186/1297-9686-42-6

**Published:** 2010-02-25

**Authors:** Joaquim Tarrés, Marta Fina, Jesús Piedrafita

**Affiliations:** 1 Grup de Recerca en Remugants, Departament de Ciència Animal i dels Aliments, Universitat Autònoma de Barcelona, 08193 Bellaterra, Spain

## Abstract

**Background:**

A procedure to measure connectedness among herds was applied to a beef cattle population bred by natural service. It consists of two steps: (a) computing coefficients of determination (CDs) of comparisons among herds; and (b) building sets of connected herds.

**Methods:**

The CDs of comparisons among herds were calculated using a sampling-based method that estimates empirical variances of true and predicted breeding values from a simulated n-sample. Once the CD matrix was estimated, a clustering method that can handle a large number of comparisons was applied to build compact clusters of connected herds of the Bruna dels Pirineus beef cattle. Since in this breed, natural service is predominant and there are almost no links with reference sires, to estimate CDs, an animal model was used taking into consideration all pedigree information and, especially, the connections with dams. A sensitivity analysis was performed to contrast single-trait sire and animal model evaluations with different heritabilities, multiple-trait animal model evaluations with different degrees of genetic correlations and models with maternal effects.

**Results:**

Using a sire model, the percentage of connected herds was very low even for highly heritable traits whereas with an animal model, most of the herds of the breed were well connected and high CD values were obtained among them, especially for highly heritable traits (the mean of average CD per herd was 0.535 for a simulated heritability of 0.40). For the lowly heritable traits, the average CD increased from 0.310 in the single-trait evaluation to 0.319 and 0.354 in the multi-trait evaluation with moderate and high genetic correlations, respectively. In models with maternal effects, the average CD per herd for the direct effects was similar to that from single-trait evaluations. For the maternal effects, the average CD per herd increased if the maternal effects had a high genetic correlation with the direct effects, but the percentage of connected herds for maternal effects was very low, less than 12%.

**Conclusions:**

The degree of connectedness in a bovine population bred by natural service mating, such as Bruna del Pirineus beef cattle, measured as the CD of comparisons among herds, is high. It is possible to define a pool of animals for which estimated breeding values can be compared after an across-herds genetic evaluation, especially for highly heritable traits.

## Background

The best linear unbiased prediction (BLUP) of breeding values allows meaningful comparisons between animals, but only when genetic links exist between the different environments (e.g. [1]). Connectedness, in a statistical sense, relates to the estimability of all contrasts involving fixed-model effects [2]. However, connectedness is not required in order to predict random breeding values [3], and disconnected subsets of records do not lead to biased predictions of breeding values so long as breeding values of base animals (i.e. the animals present at the start of performance recording) are distributed randomly and identically across the entire population [4]. This assumption is violated, however, if selection or genetic drift occurs before pedigree and performance recording begin and cause genetic means of the herds to differ [5]. The isolated herds (not highly connected i.e. for which the accuracy of comparison is low) are likely to have different genetic means. In such a case, the environment and genetic effects are partially confounded and the genetic differences between animals in different environments are underestimated. Laloë and Phocas [6] have shown that decreases in both accuracy and potential bias in a genetic evaluation are due to this phenomenon of regression towards the mean.

Laloë [7] has defined disconnectedness for random effects in terms of "non-predictability" of contrasts: a contrast is not predictable if its coefficient of determination (CD) is null. Several other methods developed to evaluate connectedness have been based on prediction error (co)variances (e.g., [7-9]). The prediction error variance (PEV) of a contrast of mean differences can be obtained using matrix absorption [10] and has a strong relationship with CD; it is thus a potential alternative measure of connectedness. These statistics have been used to measure connectedness in dairy cattle [11], swine [9,12,13], and beef cattle [14]. However, CD was found to combine data structure and amount of information better [15]. It also provides a balance between the decrease of PEV and the loss of genetic variability due to genetic relationships between animals. Laloë et al. [15] have concluded that CD was the best method for judging the precision of a genetic evaluation or optimising corresponding designs, especially when genetic relationships among animals are to be accounted for through a relationship matrix. However, CD is difficult to calculate for routine genetic evaluation due to storage and the processing time required to calculate the inverse of the coefficient matrix and the (non-inverted) relationship matrix [5]. Kuehn et al. [5] have advocated measuring connectedness using other criteria, highly correlated to CD, but easier to compute. Another way to circumvent this drawback is to turn to methods of approximated estimation of variance-covariance matrices. Garcia-Cortes et al. [16] and Fouilloux and Laloë [17] have proposed sampling methods that, theoretically, allow the estimation of entire variance-covariance matrices, and, as a result, the estimation of the CD of contrasts among genetic levels of herds. Based on these methods, Fouilloux et al. [18] have described a new two-step process to analyze connectedness among herds: the first step involves computing the CD of comparisons between groups of animals using a sampling method, while in the second step, clusters of well-connected groups are formed based on a "criterion of admission to the group of connected herds" (CACO) that reflects the level of connectedness of each herd. The procedure accounts for known pedigree and data structure efficiently when measuring connectedness among herds. This clustering method was appropriate in condensing the relevant information of large matrices of similarities (here, the CD of contrasts between genetic levels of herds). It meets the requirement to construct sets of well-connected herds, and may handle large problems very quickly [18].

This method was applied by Fouilloux et al. [18] to beef cattle breeds that use artificial insemination. In this case, links between herds come through reference sires that have progeny in different herds and a sire model can be sufficient to establish connectedness among herds. However, in many local beef cattle breeds, natural service is almost exclusively used. In this case, links due to reference sires are not so important and it is necessary to consider the connection due to maternal and paternal grandsires [19]. Thanks to the simplicity of the CACO method, different models of analysis may be easily adapted to account for these connections [18]. The choice of the best model for the sampling method depends on the size of the analyses and the knowledge of the pedigree. Hence, application of single- or multi-trait analyses using an animal model with or without maternal effects will be possible for small-sized evaluations, while sire or sire-maternal grandsire models can be used for large-sized evaluations, depending on the number of unknown sires or grandsires in the pedigree files [18].

Bruna dels Pirineus is a local beef breed selected from the old Brown Swiss (derived from the Canton Schwyz), which is similar to the American Braunvieh. The herds are located in the Pyrenean mountain areas of Catalonia (Spain). Genetic differences among beef herds are likely. Herd sizes are generally small, relative to other livestock species, and artificial insemination (AI), an effective tool for connecting herds of other beef and dairy cattle, is practically nonexistent in this breed. In contrast to other countries, cooperative breeding schemes, designed to create such genetic links [6], have been rarely used in Spain.

The objective of this study was to measure the connectedness among herds of beef cattle bred by natural service. In particular, the CD of comparisons between Bruna dels Pirineus herds will be computed using a sampling method based on an animal model and clusters of well-connected herds will be formed. This study should permit the determination of the risk of bias when comparing and selecting animals from different herds on estimated breeding values (EBV), and the results obtained can then be used as a reference for other beef cattle breeds, which are almost exclusively bred by natural service.

## Materials and methods

### Data

Data of the on-farm beef cattle evaluation for the Bruna dels Pirineus breed were used in this study. The dataset consisted of 28546 records and the total number of animals in the pedigree file was 35546. The genetic evaluation model was an animal model that included sex (2 levels), parity (10 levels), twins (2 levels), herd effect (76 levels), month (12 levels) and year (26 levels) as fixed effects. The connectedness was studied among the 76 herds that had calf performances recorded during the last five years.

### Estimation of CD of contrasts

The method presented by Fouilloux and Laloë [17] to estimate CD of estimated breeding values in a sire model has been applied to an animal model to approximate the CD of contrasts between herds. The procedure is as follows:

1- Starting from the pedigree of the population, the animals involved in the simulation are sorted from the oldest to the youngest. An animal model, including pedigree with full relationships, was used for the simulation. The same one was used in the EBV prediction model.

2- The direct genetic value *u*_*i *_of the animal i is calculated according to the status of its sire (j) and dam (k). If j and k are unknown, *u*_*i *_is generated from . If j is known and k is unknown, *u*_*i *_is calculated by *u*_*i *_= 0.5*u*_*j *_+ *φ*_*i *_where *φ*_*i *_is drawn from . The same if k is known and j is unknown, *u*_*i *_is calculated by *u*_*i *_= 0.5*u*_*k *_+ *φ*_*i *_where *φ*_*i *_is drawn from . Finally, if j and k are both known, *u*_*i *_is calculated by *u*_*i *_= 0.5(*u*_*j *_+ *u*_*k*_) + *φ*_*i *_where *φ*_*i *_is drawn from .

3- Performance of each performance-tested animal *y*_*i *_= *h*_*i *_+ *u*_*i *_+ *e*_*i *_was simulated using its generated breeding value *u*_*i *_and a residual *e*_*i *_drawn from . Herd effects *h*_*i *_were simulated multiplying a value drawn from *U*[0,1] by twice the phenotypic standard deviation. The remaining fixed effects were set to 0.

4- The vector of BLUP estimated breeding values  is obtained by solving the mixed model equations using **y**. BLUP was estimated using PEST software, ceasing iteration when the convergence criterion was less than 10^-6^. This process repeated n times leads to vectors of true (simulated) {**u**_*k*_}_*k *= 1, *n *_and estimated breeding values .

5- The CD of contrasts of interest are estimated by computing their empirical variances and covariances (quoted with *) following Fouilloux et al. [18]:

with

and

Typically, a given contrast can be written as a linear combination of the breeding values (**c'u**). For instance, on one hand, the CD of the breeding value of a single animal (i.e. its reliability) is obtained by using a vector **c' **null except a 1 in the appropriate position corresponding to this breeding value. On the other hand, the CD of contrasts among herds i and j is obtained by using a vector **c' **null except a  or a  in the appropriate position corresponding to animals from herd i and j respectively. Here, *m*_*i *_and *m*_*j *_were respectively the number of animals in herd i and j.

The estimated values of the CD of comparison among herds were computed by performing 1000 replicates of the re-sampling method.

### Selecting the set of connected herds

The main practical goal of connectedness studies is to identify sets of connected herds. Two herds are considered connected if its CD is greater than an *a priori *threshold, say χ. A set of connected herds should then be built in such a way that any pairwise CD between herds of the set is greater than χ. This was achieved through an agglomerative clustering procedure proposed for Fouilloux et al. [18], which was designed explicitly for building compact clusters and is suitable for large-sized datasets. At the start of the process, each herd begins in a cluster by itself, and each step involves aggregating herds one by one into appropriate clusters:

1. Each herd begins in the cluster by itself: [{*h*_1_},{*h*_2_}, ..., {*h*_*n*_}]. The two herds linked by the highest CD, say *h*_1 _and *h*_2_, are clustered together, leading to the following partition: [{*h*_1_, *h*_2_}, ..., {*h*_*n*_}].

2. A similarity index is calculated for each herd outside the cluster {*h*_1_, *h*_2_}. The similarity index of a given herd is equal to its lowest CD with the herds currently in the cluster. The herd with the highest similarity index is added to the cluster. The CACO of this new clustered herd is equal to its similarity index at this step. Supposing, for the sake of simplicity, that this herd is {*h*_3_}, then, the new partition is the following: [{*h*_1_, *h*_2_, *h*_3_}, ..., {*h*_*n*_}].

The process stops either when all herds are clustered, or when the CD of comparison between the clustered herds and each of the remaining herds are all below the fixed *a priori *threshold χ. In that latter case, the algorithm is applied to the remaining herds to build other possible clusters. Finally, two herds within the same cluster are ensured to be compared with a CD > χ.

When applying this method, a decision needs to be made on the threshold χ for the CD to be achieved before a herd is considered to be connected. Such a decision is and will always be a subjective matter. The threshold χ was chosen to be equal to 0.4, as in Fouilloux et al. [18]. However, a more informed choice is possible using CD as a criterion of accuracy and potential bias, and by considering the relationships between CD, the amount of information, and the quality of design.

### Sensitivity analysis

For the sensitivity analysis, three different heritabilities were simulated, first representing low (0.10), moderate (0.25) and high (0.40) genetic variations. Second, the results of an animal model were compared with results from a sire model. In such a case, the data were simulated using an animal model with pedigree but the genetic evaluation was done using a sire model. Here, two models were evaluated: (i) the sire model does not take into account the pedigree, i.e. the sire effects follow a  where  was a quarter of the genetic variance, and (ii) the sire model includes a pedigree, i.e. the sire effects follow a  where  was a quarter of the genetic variance and **A**_*s *_was the relationship matrix of sires.

Third, the estimation of CD was implemented for multi-trait animal models where the genetic values were simulated in Step 2 as **u **= [**u**_**l**_, **u**_**2**_] ~ *MVN*(**0,G**) and the residual values were simulated in Step 3 as **e **= [**e**_**l**_, **e**_**2**_] ~ *MVN*(**0,R**). The genetic and residual (co)variance matrices were respectively:

Two different multi-trait scenarios were simulated: (i) a lowly heritable trait (0.10) with a moderate negative genetic correlation (-0.25) and moderately heritable trait (0.40); and (ii) a lowly heritable trait (0.10) with a high negative genetic correlation (-0.50) and highly heritable trait (0.40). First, these two scenarios were simulated with a null residual correlation but, as a null residual correlation was not always realistic, the effect of a non-null residual correlation was checked by simulating residual correlations with the same magnitude of the genetic correlations. The simulated data were analyzed jointly in Step 4, but the CDs were estimated separately for each trait in Step 5.

Fourth, the estimation of CD was implemented for models with maternal effects, where the direct and maternal genetic values were simulated in Step 2 as [**u m**] ~ *MVN*(**0,G**). The genetic and residual (co)variance matrices were, respectively:

Two different scenarios with maternal effects were simulated: (i) a trait with a lowly heritable maternal effect (0.10), moderate negative genetic correlation (-0.25) and moderately heritable direct effect (0.25), and (ii) a trait with lowly heritable maternal effect (0.10), high negative genetic correlation (-0.50) and highly heritable direct effect (0.40). Both scenarios were compared in the case of a null genetic correlation among maternal and direct effects. In Step 3, the performance of each performance-tested animal *y*_*i *_= *h*_*i *_+ *u*_*i *_+ *m*_*k *_+ *e*_*i *_was simulated using the herd effect *h*_*i*_, its generated direct breeding value *u*_*i*_, the maternal breeding value of its dam *m*_*k *_and a residual *e*_*i *_drawn from . The simulated data were analyzed using a model with maternal effects in Step 4, but the CDs were estimated separately for the direct and maternal effect in Step 5.

## Results

### Individual reliabilities

First, the sampling method to estimate CD (reliabilities) of estimated breeding values was applied to an animal model. The mean reliability of the 28546 animals with data decreased from 0.51 to 0.22 as the heritability decreased from a high (0.40) to a low (0.10) value (Table 1). This reliability was 0.37, with a standard deviation of 0.08 when the simulated heritability was 0.25. The reliability of sires in the first breeding season (with 0 to 30 progeny) was under the minimum reliability determined by Interbull [20] to publish bull indexes (0.50-0.75). This reliability became sufficiently high for publication of breeding values after the first breeding season, i.e. 0.69 for sires with 30 to 60 progeny, and increased up to 0.86 for sires with over than 150 progeny (Table 1). The reliabilities of sires were 0.07 to 0.09 points higher with an animal model than with a sire model, although they increased only between 0.01 and 0.03 points if the pedigree is not taken into account in the sire model. These differences were lower for the lowly heritable traits and increased for the highly heritable traits.

**Table 1:** Average reliabilities of individual animals in single trait evaluations with different heritabilities (h^2^)

**h**^**2**^	Model	Animals with data	Sires with progeny						Dams
				
			0-30	30-60	60-90	90-120	120-150	>150	
0.40	Sire nr^1^		0.38	0.68	0.72	0.74	0.74	0.90	
	Sire		0.40	0.69	0.74	0.75	0.75	0.91	
	Animal	0.51	0.49	0.79	0.84	0.85	0.83	0.91	0.26

0.25	Sire nr		0.30	0.60	0.66	0.69	0.69	0.86	
	Sire		0.32	0.62	0.68	0.70	0.70	0.87	
	Animal	0.37	0.39	0.69	0.76	0.79	0.77	0.86	0.18

0.10	Sire nr		0.17	0.42	0.51	0.55	0.57	0.74	
	Sire		0.19	0.45	0.53	0.57	0.58	0.77	
	Animal	0.22	0.23	0.48	0.58	0.63	0.63	0.75	0.09

Number		28546	364	97	52	22	17	23	6354

^1 ^Sire nr: sire model without relationship

In the multiple trait scenario with a null residual correlation, the mean reliability of the 28546 animals with data on lowly heritable traits increased from 0.22 to 0.23 and 0.29 in the multiple trait models with moderate (-0.25) and high (-0.50) genetic correlation respectively (Table 2). The increase in reliability was higher as reliability of the animal decreased. However, these gains were not so important when the magnitude of the residual correlation was equal to the genetic correlation (Table 2).

**Table 2:** Average reliabilities for the lowly heritable trait (h^2 ^= 0.10) of individual animals in multiple trait evaluations

**Model**^**1**^			Animals with data	Sires with progeny						Dams
			
**h**^**2**^	**r**_**g**_	**r**_**e**_		0-30	30-60	60-90	90-120	120-150	>150	
ST			0.22	0.23	0.48	0.58	0.63	0.63	0.75	0.09
0.25	-0.25	-0.25	0.22	0.23	0.50	0.58	0.64	0.63	0.75	0.09
0.25	-0.25	0	0.23	0.24	0.50	0.59	0.65	0.63	0.76	0.10
0.40	-0.5	-0.5	0.25	0.25	0.51	0.59	0.65	0.64	0.76	0.11
0.40	-0.5	0	0.29	0.29	0.54	0.61	0.67	0.65	0.77	0.13

Number			28546	364	97	52	22	17	23	6354

^1 ^Trait evaluated jointly with another trait with heritability (h^2^) and genetic correlation (r_g_) and residual correlation (r_e_) except in the single trait evaluation (ST)

In models with maternal effects, reliabilities of the animals for the direct effects were similar to those obtained from single-trait evaluations (results not shown); in particular, the reliability of dams for maternal effects was 0.21. This reliability increased if a genetic correlation with the direct effects existed. The increase was equal to 0.04 point if the genetic correlation was high (-0.5) with a highly heritable trait (0.40) (Table 3). However, the reliability only became high enough to publish breeding values for maternal grandsires with more than 30 dam progeny (Table 3).

**Table 3:** Average reliabilities for the maternal effects (h^2 ^= 0.10) of individual animals in single trait evaluations with maternal effects

**Model**^**1**^		Animals with data	Sires with progeny						Dams	MGS	
				
**h**^**2**^	**r**_**g**_		0-30	30-60	60-90	90-120	120-150	>150		0-30	30-60
0.25	0	0.14	0.14	0.21	0.26	0.40	0.34	0.50	0.21	0.23	0.72
0.25	-0.25	0.13	0.15	0.25	0.30	0.43	0.37	0.53	0.22	0.24	0.73
0.40	0	0.15	0.14	0.23	0.28	0.42	0.36	0.51	0.20	0.24	0.72
0.40	-0.5	0.15	0.19	0.35	0.40	0.51	0.45	0.59	0.25	0.29	0.76

Number		28546	364	97	52	22	17	23	6354	345	12

^1 ^The direct effects had heritability (h^2^) with genetic correlation (r_g_) with maternal effects

### CD of comparisons between herds

Once the 76 × 76 matrix of CD of contrasts among herds was estimated, the average CD per herd was calculated as the mean of the 76 CD values of each herd column. Later on, mean, standard deviation, minimum and maximum of the 76 average CD per herd were calculated. The mean of average CDs per herd in the single-trait animal model decreased from 0.53 to 0.31 as the simulated heritabilities decreased from 0.40 to 0.10. The percentage of herds contrasts with CD higher than 0.4 decreased with the heritability from 85.93% to 25.54% (Table 4).

**Table 4:** Average coefficients of determination (CD) of contrasts per herd in single trait evaluations with different heritabilities (h^2^)

**h**^**2**^	Model	Average CD				% CD over 0.4
			
		Mean	STD^2^	Minimum	Maximum	
0.40	Sire nr^1^	0.260	0.108	0.068	0.509	19.96
	Sire	0.285	0.111	0.074	0.534	23.66
	Animal	0.535	0.086	0.302	0.705	85.93

0.25	Sire nr	0.220	0.098	0.057	0.464	13.81
	Sire	0.244	0.102	0.063	0.492	16.62
	Animal	0.455	0.087	0.243	0.644	70.70

0.10	Sire nr	0.147	0.075	0.038	0.358	4.44
	Sire	0.169	0.080	0.043	0.390	6.15
	Animal	0.310	0.079	0.144	0.512	25.54

^1 ^Sire nr: sire model without relationship.

^2 ^STD: standard deviation

The average CD per herd ranged between 0.243 and 0.644 when the simulated heritability was 0.25, with a mean of 0.455 and a standard deviation of 0.087 (Table 4). This average CD was about double than that obtained using a sire model with unknown and known pedigree (0.22 and 0.24, respectively). The percentage of connected herds was also much higher with an animal model (70.70%) than with a sire model (16.62%). The percentage of connected herds using a sire model was very poor even for highly heritable traits (Table 4), while, the degree of connection evaluated with an animal model was important for moderately and highly heritable traits but still poor for lowly heritable traits.

In the multiple trait scenario with a null residual correlation, the mean of the approximated CD of contrast for the lowly heritable traits increased from 0.31 in the single-trait evaluation to 0.35 in the multi-trait evaluation with a high genetic correlation and highly heritable trait, increasing the percentage of connected herds from 25.54% to 34.03% (Table 5). However, the increase in the percentages was not so high if there was residual correlation with the same magnitude as the genetic correlation.

**Table 5:** Average coefficients of determination (CD) of contrasts per herd for the lowly heritable trait (h^2 ^= 0.10) in multiple trait evaluations

**Model**^**1**^			Average CD				% CD over 0.4
	
h^2^	r_g_	r_e_	Mean	STD^2^	Minimum	Maximum	
ST			0.310	0.079	0.144	0.512	25.54
0.25	-0.25	-0.25	0.310	0.079	0.160	0.489	22.94
0.25	-0.25	0	0.319	0.078	0.176	0.506	24.59
0.40	-0.5	-0.5	0.325	0.078	0.157	0.498	26.60
0.40	-0.5	0	0.354	0.077	0.195	0.541	34.03

^1 ^Trait evaluated jointly with another trait with heritability (h^2^) and genetic correlation (r_g_) and residual correlation (r_e_) except in the single trait evaluation (ST)

^2 ^STD: standard deviation

In models with maternal effects, the average CD per herd for the direct effects were similar to those obtained from single-trait evaluations (results not shown), but the average CD for maternal effects were lower than in the single-trait evaluation, i.e. 0.19 vs. 0.31 respectively (Table 6). The percentage of connected herds for maternal effects was very low, less than 10% (Table 6). The mean of average CD per herd increased from 0.202 to 0.251 if the maternal effects had a high genetic correlation with the direct effects, but the percentage of connected herds only increased from 8.25% to 11.82% (Table 6).

**Table 6:** Average coefficients of determination (CD) of contrasts per herd in single trait evaluations with maternal effects

**Model**^**1**^		Average CD				% CD over 0.4
	
h^2^	r_g_	Mean	STD	Minimum	Maximum	
0.25	0	0.189	0.084	0.047	0.438	7.75
0.25	-0.25	0.203	0.082	0.054	0.461	8.21
0.40	0	0.202	0.082	0.058	0.445	8.25
0.40	-0.5	0.251	0.079	0.099	0.505	11.82

^1 ^The direct effects had heritability (h^2^) with genetic correlation (r_g_) with maternal effects

^2 ^STD: standard deviation

### Set of connected herds

**Figure 1:**
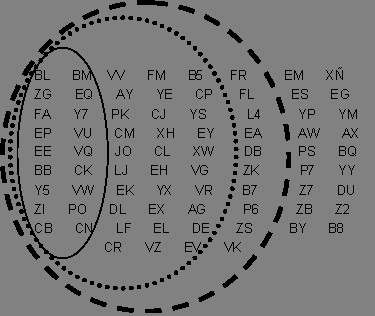
**Clusters obtained using the CACO method in single trait analysis with different heritabilities**. The heritabilities used were h^2 ^= 0.10 (Thin black line), h^2 ^= 0.25 (dotted black line) and h^2 ^= 0.50 (thick dashed line).

The clustering procedure was applied to the 76 × 76 matrix of CD of contrasts among herds. In the moderate heritability scenario (0.25), a big cluster was found including 48 herds (Figure 1). Two more clusters were found by grouping two and three herds. The rest of the herds up to 76 could not be included in any cluster. The number of herds in the big cluster was even bigger (up to 58) when the simulated heritability was high (0.40) (Figure 1). However, the number dropped to 18 herds for low heritabilities (0.10), although it still contained the larger herds of the breed because a higher number of animals per herd allowed a better comparison of the genetic level among herds.

## Discussion

The BLUP of breeding values allows comparisons between animals if the reliability is high enough, but the individual reliability is not a sufficient measure of risk in comparing animals across herds, and does not reflect potential bias in models that exclude genetic groups or increased error associated with fitting genetic groups [5]. A better criterion to assess this risk is the CD of comparisons between animals (or groups of animals) from different herds [5]. Generally, a low CD corresponds to a contrast estimated without accuracy due to some confusion between environmental and genetic differences [7]. The CD of comparisons depends on three factors: (1) the amount of information, through the number of progeny per herd; (2) the quality of the design through the proportion of progeny from reference sires within a herd; and (3) the heritability [6]. In this study, the CDs of comparisons between herds of beef cattle bred by natural service have been computed using a sampling method. These CDs were low when the genetic evaluation was done using a sire model, even for highly heritable traits. When the simulated heritability was 0.25, the mean of average CD per herd in the Bruna dels Pirineus breed (0.244) using a sire model was slightly lower than that found by Fouilloux et al. [18] in the Bazadais breed (0.294) and much lower than that of the Charolais breed (0.54). These two beef cattle breeds use artificial insemination. In these cases, links between herds come through reference sires that have progeny in different herds and a sire model can be sufficient to establish connectedness among herds. However, in many local beef cattle breeds, breeding is performed almost exclusively by natural service. The Bruna dels Pirineus breeders had never attempted a formal exchange of bulls among herds, although some amount of exchange is believed to have taken place through purchases of bulls from prominent breeders and at national shows and auctions. Because of the lack of artificial insemination and of an active exchange program, connectedness was expected to be more limited in the Bruna dels Pirineus breed than in the Bazadais breed and, especially, the Charolais breed.

The reliability of comparisons among herds increased using an animal model because more pedigree information was added, especially the connections due to maternal and paternal grandsires. In the Bruna dels Pirineus breed, Tarres et al. [19] found that the genetic similarity of connected herds was higher through maternal grandsires and paternal grandsires (25.91% and 38.91%, respectively) than through sharing sires (20.87%). As a result of including this pedigree information, the degree of connection evaluated with an animal model in the Bruna dels Pirineus breed was considerably high for moderately and highly heritable traits. However, the connectedness levels for lowly heritable traits, e.g. functional traits, were still poor.

Connectedness in genetic evaluations for lowly heritable traits can be improved by performing joint evaluations with more heritable and highly correlated traits, especially if the residual correlation among these traits is nearly null. Our results agree with Schaeffer [21], in the sense that the capacity of a multiple trait analysis to increase CD depends on residual and genetic correlations used for the analysis. First, the percentage increment of CD was dependent on the difference between error and genetic correlations. The greater the absolute difference in correlations, the greater the increment of CD for both traits [21]. Second, when the residual correlation is less (greater) than the genetic correlation, in absolute terms, then the trait with the lower (higher) heritability achieves the greatest percent increment of CD [21].

For traits with direct and maternal effects, the CDs of comparisons among herds were considerably high for direct effects. In the case of maternal effects, they can be better evaluated if a high genetic correlation exists with the direct effects. This favors the evaluation of the maternal effects for birth weight that had a heritability of 0.10 and a high negative genetic correlation (-0.5) to the highly heritable direct effect (0.40) [22]. For weaning weight, the maternal effects had a low heritability of 0.10 and a moderate negative genetic correlation (-0.25) to the moderately heritable direct effect (0.25) [22]. However, even if high genetic correlation is used in the evaluation, the comparisons among herds for maternal effects had a low reliability.

As a result of these links, most of the herds of the Bruna dels Pirineus breed were well connected, especially for moderately and highly heritable traits. The herds of this breed were located primarily within the same region: the Pyrenean area of Catalonia (Spain). Because almost all of the matings in this beef population were by natural service, the close proximity of these herds has made bulls' and heifers' exchanges more feasible. Furthermore, because they are a one-purpose breed raised for meat production, Bruna dels Pirineus breeders participating in the YRS have similar breeding objectives, creating the potential for many herds to purchase and to use related individuals. This can explain the fact that many of the herds were well connected. According to the results of the connectedness study and although all performances must be included in the genetic evaluation, only genetic values of animals coming from connected herds should be published at a "racial level," while genetic values of animals coming from disconnected herds should be used only within herds or provided with a warning that comparisons between poorly connected herds may be biased. By using sires from well-connected YRS herds, the disconnected herds should, quickly, become strongly connected with other Bruna dels Pirineus herds in the YRS. New herds entering the YRS can, therefore, become rapidly connected to the entire breed by purchasing sires from herds that are already well connected. Exchange of bulls and purchase of bulls from other herds can increase connectedness effectively and reduce the risk of bias when EBVs of animals from different herds are compared [23].

## Conclusions

The own dynamics of a beef cattle population bred by natural service could imply an important exchange of breeding animals between herds (connections) that could explain the high CD of comparisons found among herds. It was worthwhile to use an animal model when performing the sampling method to estimate the CD because adding pedigree information and, especially, considering the connections due to the dams, increased the CD values. Connectedness in genetic evaluations for lowly heritable traits can be improved by performing joint evaluations with more heritable traits with a high genetic correlation. Maternal effects can also be evaluated better if a high genetic correlation with direct effects exists. As a result of these links, most of the Bruna dels Pirineus herds were well connected and the genetic evaluation will allow producers to identify breeding animals that are potentially better than their own, especially for moderately and highly heritable traits. The genetic values of animals coming from connected herds should be published at a "racial level," while genetic values of animals coming from disconnected herds should be used only within herds or provided with a warning that comparisons between poorly connected herds may be biased.

## List of abbreviations used

BLUP: best linear unbiased prediction; CACO: criterion of admission to the group of connected herds; CD: coefficient of determination; EBV: estimated breeding values; YRS: yield recording scheme.

## Competing interests

The authors declare that they have no competing interests.

## Authors' contributions

JT performed the statistical analysis and drafted the manuscript. MF managed the YRS of the Bruna dels Pirineus breed and revised the manuscript critically for important intellectual content. JP supervised the YRS, promoted the study and revised the manuscript critically for important intellectual content. All authors read and approved the final manuscript for authors.
